# High frequency of azole resistant *Candida spp*. colonization among presumptive multidrug resistant tuberculosis (MDR-TB) patients

**DOI:** 10.1371/journal.pone.0242542

**Published:** 2020-11-19

**Authors:** Surya Darma, Angga Ambara, Abu Tholib Aman, Luthvia Annisa, Nurrokhman, Titik Nuryastuti, Tri Wibawa

**Affiliations:** Department of Microbiology, Faculty of Medicine Public Health and Nursing, Universitas Gadjah Mada, Yogyakarta, Indonesia; Rutgers Biomedical and Health Sciences, UNITED STATES

## Abstract

**Background:**

Tuberculosis is one of the major causes of death globally. The problems become even more complicated with the rise in prevalence of multidrug resistant tuberculosis (MDR-TB). Many diseases have been reported to occur with tuberculosis making it more difficult to manage. *Candida spp*., which are yeast-like fungi and a constituent of normal flora in humans, are notoriously reported to be one of the most common opportunistic nosocomial infections. This study aimed to measure the proportion of presumptive MDR-TB patients colonized with *Candida spp*. and to characterize its susceptibility against azole group antifungal agents.

**Methods:**

Sputum from presumptive MDR-TB patients were collected and examined for the presence of *Mycobacterium tuberculosis* and its rifampicin resistant status using GeneXpert. It was further cultured on *Sabouroud’s Dextrose Agar* (SDA) to isolate the *Candida spp*. The Candida species were determined using *HiCrome™ Candidal Differential Agar*. Antifungal susceptibility was tested using microbroth dilution methods. Checkerboard microdilution assays were performed to measure the interaction between rifampicin and fluconazole to *C*. *albicans*.

**Results:**

There were 355 presumptive MDR-TB patients enrolled. A total of 101 (28.4%) patients were confirmed to have *M*. *tuberculosis*. There were 113 (31.8%) sputum positive for *Candida spp*., which corresponded to 149 *Candida spp*. isolates. *Candida albicans* was the most frequent (53.7%) species isolated from all patients. The susceptibility of *Candida spp*. against fluconazole, itraconazole, and ketoconazole were 38.3%, 1.3%, and 10.7% respectively. There was significant association between rifampicin exposure history and susceptibility of *Candida albicans* against fluconazole (Odds Ratio: 9.96; 95% CI: 1.83–54.19; p <0.01), but not for ketoconazole and itraconazole. The checkerboard microdilution assays showed that rifampicin decreased the fungicidal activity of fluconazole to *C*. *albicans* in a dose-dependent manner.

**Conclusion:**

There was high frequency of azole resistant *Candida spp*. isolates colonizing the respiratory tract of presumptive MDR-TB patients. This presence might indicate the association of chronic exposure to rifampicin, the main drug for tuberculosis therapy, with the induction of azole resistance.

## Introduction

Tuberculosis (TB) is an infectious disease caused by rod shaped bacteria called *Mycobacterium tuberculosis*. The disease usually affects the lungs, but it can also involve other parts of the human body. Globally, TB is one of the top 10 causes of death. Approximately 1.2 million deaths among HIV-negative people were due to TB and an estimated 10 million people developed TB in 2018. TB has become a global problem due to the emergence of *M*. *tuberculosis* strains which are resistant to many anti-tuberculosis drugs. These multidrug resistant tuberculosis (MDR-TB) strains are more difficult to treat and control. Rifampicin is one of the main drugs in TB therapy and is a key indicator of the emergence of MDR-TB strains [1].

Despite its catastrophic effect as a single disease, many diseases have been reported to occur with TB making it more difficult to manage. Patients with TB may present with several additional infections such as HIV-AIDS [2], other bacterial infections (caused by *Klebsiella* and *Pseudomonas*) [3], malaria [4], histoplasmosis [5], and candidiasis [6].

*Candida spp*. are yeast-like fungi and a constituent of normal flora in humans. It frequently thrives on human skin, the gastrointestinal tract, the genitourinary tract of women and the respiratory tract [7]. As normal flora in the respiratory tract, *Candida spp*. are rarely reported as significant causative agents of infection. In the clinical and laboratory diagnosis setting, it is difficult to define conditions when *Candida spp*. are detected in the respiratory tract. It is hard to judge the existence of *Candida spp*. as a contaminant, commensal, colonizer, or infection [8]. However, *Candida spp*. were notoriously reported as one of the most common opportunistic nosocomial fungal infections [9]. As a single contagion, it causes several serious diseases including vulvovaginal candidiasis [10], oral candidiasis [11], and other invasive fungal infections [12].

There are several pathogenetic roles of *Candida spp*. colonization in the respiratory tract. *Candida spp*. are not innocent flora which normally colonize the respiratory tract. It may contribute to the occurrence of bacterial infections such as pneumonia in animal models through the deterioration of the host immune response. *Candida spp*. colonization was found to elicit a Th1-Th17 immune response that supports the development of bacterial pneumonia via inhibition of bacterial phagocytosis by alveolar macrophages [13]. *Candida spp*. colonization was also reported to increase mortality rate and is associated with increased rate of drug resistance in patients with ventilator associated pneumonia (VAP) [14]. The role of *Candida spp*. in the respiratory tract either in colonization or infection conditions involves a complex interaction of host immune response, *Candida spp*. virulence factors, and the presence of other bacterial and fungal pathogens [8, 15–17].

Many reports mentioned the effect of *Candida spp*. to the bacterial causative agent of respiratory tract infection, but there is very limited data concerning the impact of bacterial pathogens on the *Candida spp*. itself. In addition, there are several studies reporting the colonization of *Candida spp*. in patients with TB but there is no report that showed the interaction of TB and candida colonization or infection [6, 18–20]. Here, the authors report the frequency of *Candida spp*. colonization among presumptive MDR-TB patients and the possible influence of rifampicin, one of the most powerful anti-mycobacteria agents, on the susceptibility of *Candida albicans* against fluconazole, the drug of choice for Candidiasis caused by *C*. *albicans*.

## Materials and methods

### Patients and sputum collection

The study protocol of this laboratory observational study was approved by The Medical and Health Research Ethics Committee of Faculty of Medicine Public Health and Nursing Universitas Gadjah Mada / Dr. Sardjito General Hospital (Reference number: KE/FK/0502/EC/2018). Informed consent was waived by ethic committee. Sputum from presumptive MDR-TB patients sent to the Laboratory of Tuberculosis, Department of Microbiology, Faculty of Medicine Public Health and Nursing, Universitas Gadjah Mada from September–December 2016 were collected. The patient’s sputum samples were sent to the laboratory to confirm TB in patients diagnosed with the presumptive presence of MDR-TB. The patients must fulfil one or more out of nine (9) criteria of presumptive MDR-TB issued by the Indonesian Ministry of Health [21]. The following are the nine criteria of presumptive MDR-TB being tested using GeneXpert: (1) Patient failed to complete category 2 treatment; (2) Patient failed to complete category 2 treatment and no conversion during 3 months; (3) Patient experienced non-standardized TB treatment; (4) Patient failed to complete category 1 treatment; (5) Patient failed to complete category 1 treatment and no conversion; (6) Patient relapsed; (7) Patient admitted to clinic after being lost to follow up; (8) Presumptive TB patients with history of close contact with MDR-TB patients; and (9) Patient with TB-HIV co-infection who is not responding to antimycobacterial drugs treatment.

The history of rifampicin administration was obtained from the form that was completed by the attending pulmonologist. Any patient falling into criteria number 8 was considered rifampicin non-exposed, and the remaining were considered as rifampicin exposed. The sputum was labelled using unique codes which did not contain any identifiable information about the patients.

### Tuberculosis diagnosis by using GeneXpert

Sputum obtained from patients underwent TB and rifampicin resistance status detection using GeneXpert MTB/RIF (Cepheid, USA) according to the manufacturer’s protocol. Sputum samples were mixed with the provided buffer (2:1 v/v ratio), homogenized, transferred to GeneXpert cartridges, and were subsequently analysed for the presence of *M*. *tuberculosis* DNA mutations corresponding to the rifampicin resistant phenotype.

### *Candida spp*. culture and identification

Sputum were inoculated into *Sabouroud’s Dextrose Agar* (SDA) with chloramphenicol supplementation and incubated in room temperature for 24–48 hours. The yeast that grew on the medium were further identified using microscopic examination after lacto phenol cotton blue staining, germ tube test, and colonies morphology identification [22]. *Candida spp*. grown in the SDA media were isolated and inoculated into *HiCrome™ Candidal Differential Agar* (HiMedia Laboratories Pvt. Limited, India). The inoculates were incubated in 30°C for 40–48 hours. Species identification was done by observing the color of the colonies as recommended by the manufacturer. *Candida albicans* appeared light green in color, *Candida tropicalis* was blue, *Candida glabrata* was cream to white, and *Candida krusei* was purple [6, 23].

### Antifungal susceptibility testing

*Candida spp*. were subjected to anti-fungal susceptibility testing for azole group compounds, i.e. fluconazole, ketoconazole and itraconazole. The anti-fungal susceptibility testing was done according to the CLSI manual M27 S3 [24]. Briefly, 0.5 McFarland of *Candida spp*. suspension was prepared and distributed into a 96 well microplate which contained RPMI1640 medium and serially diluted antifungal agents. The plates were incubated at 35°C for 48 hours. The minimum inhibitory concentration (MIC) of the antifungals were determined, and susceptibility against antifungals were interpreted according to the CLSI manual.

### Checkerboard microdilution assay

**Fig 1:**
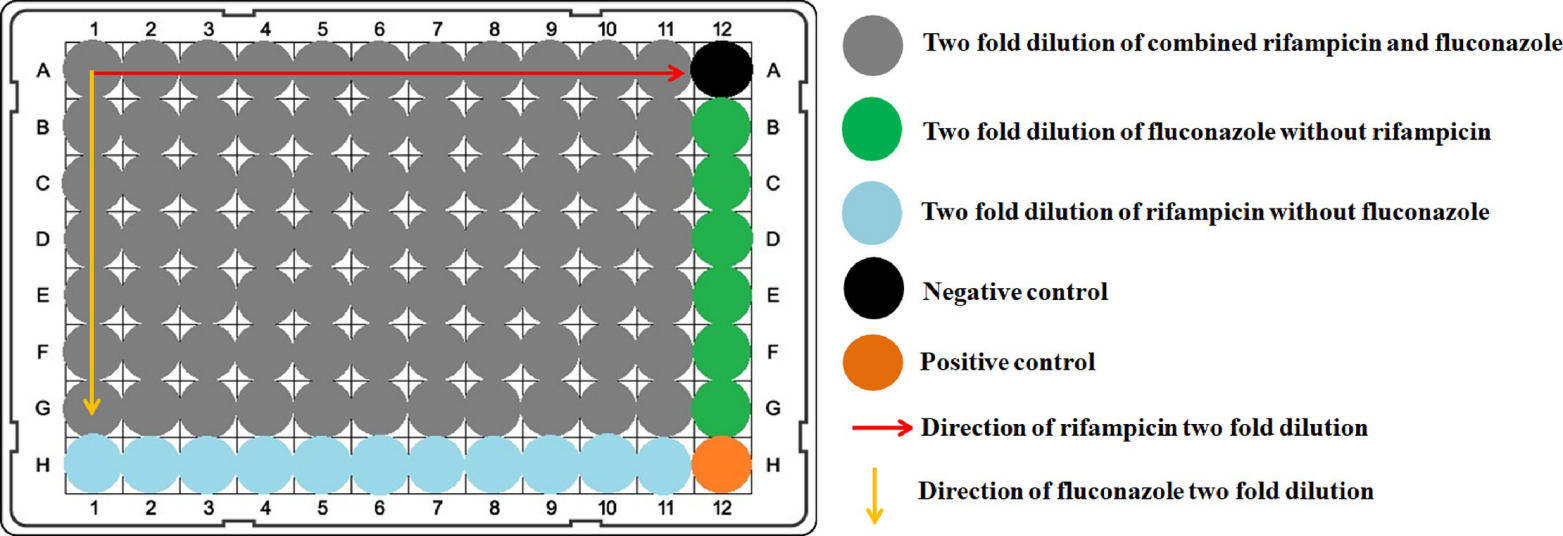
The schematic diagram of checkerboard microdilution assays. Rifampicin was serially diluted along the abscissa while fluconazole was diluted along the ordinate. Each well was inoculated with 2 μl *C*. *albicans* inoculum (0.5 McFarland). The highest concentration of fluconazole and rifampicin was in well A1 and the lowest concentration of fluconazole and rifampicin was in well G11. Well A12 was the negative control and well H12 was the positive control.

Checkerboard microdilution assay was carried out on fluconazole sensitive and resistant *C*. *albicans* isolates to test the effect of rifampicin on the fungicidal activity of fluconazole. Assays were performed according to the CLSI (M27-A) standard method using a 96 well round bottom microplate. A total of 200 μl of RPMI 1640 media, supplemented with fluconazole and rifampicin, were distributed into each well of the microplates. Rifampicin was serially diluted along the abscissa while the fluconazole was diluted along the ordinate. Each well was inoculated with 2 μl *C*. *albicans* inoculum (0.5 McFarland) (Fig 1). Final fluconazole concentrations were 0.25 μg/ml, 0.5 μg/ml, 1 μg/ml, 2 μg/ml, 4 μg/ml, and 8 μg/ml for fluconazole sensitive *C*. *albicans* and 8 μg/ml, 16 μg/ml, 32 μg/ml, 64 μg/ml, 128 μg/ml, 256 μg/ml, and 512 μg/ml for fluconazole resistant *C*. *albicans*. Final rifampicin concentrations were 0.16 μg/ml, 0.31 μg/ml, 0.63 μg/ml, 1.25 μg/ml, 2.5 μg/ml, 5 μg/ml, 10 μg/ml, 20 μg/ml, 40 μg/ml, 80 μg/ml, and 160 μg/ml. The 96 well round bottom microplates were incubated at 35°C for 48 hours under aerobic conditions. A spectrophotometer was used to measure the optical density (OD) at 620 nm. Data were obtained from triplicate individual experimentations for each *C*. *albicans* isolate.

### Statistical analysis

Two (2) sample proportion hypothesis tests were computed. Chi Square analysis was done to determine the association of rifampicin exposure and susceptibility of *C*. *albicans* against fluconazole. ANOVA tests were done to determine the significance of rifampicin effect to fluconazole activity. All analysis was done using the STATA^®^ Software (StataCorp LLC).

## Results

Sputum from 355 presumptive MDR-TB infection patients were analyzed of which 101 sputum samples (28.4%) were confirmed to be positive for TB by GeneXpert, and nine (8.9%) were found to be rifampicin resistant. It was found that 113 (31.8%) were colonized by *Candida spp*. The proportion of males was greater than females in both *Candida spp*. colonized and non-colonized groups. However, it was noted that the proportion of male patients was greater in the *Candida spp*. colonized group (p<0.05). Patients who were not confirmed infected by *M*. *tuberculosis* were usually not colonized by *Candida spp*. (p<0.05). However, there was no significant association between tuberculosis and *Candida spp*. colonization (Table 1).

**Table 1:** Characteristics of patients.

Characteristics	*Candida spp*. Colonization (n = 113)	*Candida spp*. Non-Colonization (n = 242)
Age		
<18	17	40
19–65	87	179
>65	9	23
Gender		
Male	83*	144*
Female	30*	98*
Diagnosis of TB		
GeneXpert positive TB	27	74
Rifampicin resistant positive	2	7
Rifampicin resistant negative	25	67
GeneXpert negative TB	86*	168*

* = p < 0.05

The patients sputum samples were dominantly (71.6%) colonized by a single species of *Candida*. There was a significant difference in the proportion of single and double species colonization in *M*. *tuberculosis* infected and non-infected groups (p<0.05). Single species colonization was more frequently found in the *M*. *tuberculosis* non-infected group (76.7%) compared to the infected group (55.6%). On the other hand, double species colonization was more frequently found in the *M*. *tuberculosis* infected group (40.7%) compared to the non-infected group (19.8%) (Table 2).

**Table 2:** Number species of *Candida spp*. isolated in patients.

*Candida sp* isolated from patients	GeneXpert Results				Total	
	*M*. *tuberculosis* (+)		*M*. *tuberculosis* (-)			
	n	%	n	%	n	%
1 Species	15	55.6*	66	76.7*	81	71.6
2 Species	11	40.7*	17	19.8*	28	24.8
3 Species	1	3.7	3	3.5	4	3.6
Total patients	27	100	86	100	113	100

* = p < 0.05

One hundred forty-nine (149) *Candida spp*. were isolated from 113 patients. Table 3 shows that *C*. *albicans* was the most frequent (53.7%) species isolated. Interestingly, *C*. *albicans* was more frequently found in non-TB patients (57.8%) compared to 42.5% in TB patients (p<0.05).

**Table 3:** Identification of *Candida spp*. isolates and distribution in *M*. *tuberculosis* infected and non-infected patients.

*Candida spp*.	*M*. *tuberculosis* (+)		*M*. *tuberculosis* (-)		TOTAL	
	n	%	n	%	n	%
*C*. *albicans*	17	42.5*	63	57.8*	80	53.7
*C*. *tropicalis*	10	25.0	21	19.3	31	20.8
*C*. *glabrata*	10	25.0	19	17.4	29	19.5
*C*. *krusei*	3	7.5	6	5.5	9	6.0
**TOTAL**	40	100	109	100	149	100.0

* = p < 0.05

Antifungal susceptibility test was performed on all *Candida spp*. isolated from the patients. Three (3) antifungals that belonged to the azole group was tested namely fluconazole, itraconazole, and ketoconazole. The susceptibility of *Candida spp*. against fluconazole, itraconazole and ketoconazole are 38.3%, 1.3%, and 10.7% respectively. There is no significant difference in the pattern of susceptibility to azole among the *Candida spp*. isolated from *M*. *tuberculosis* positive and negative groups (Table 4).

**Table 4:** Distribution of *Candida spp*. isolates susceptibility to antifungal agents according to the *M*. *tuberculosis* detection results.

Anti-fungal drugs	*M*. *tuberculosis* (+) patients (n = 40)	*M*. *tuberculosis* (-) patients (n = 109)	TOTAL (n = 149)
**Fluconazole**			
Susceptible	16 (40%)	41 (37.8%)	57 (38.3%)
Resistant	24 (60%)	68 (62.2%)	92 (62.7%)
**Itraconazole**			
Susceptible	0 (0%)	2 (1.8%)	2 (1.3%)
Resistant	40 (100%)	107 (98.2%)	147 (98.7%)
**Ketoconazole**			
Susceptible	2 (5%)	14 (12.8%)	16 (10.7%)
Resistant	38 (95%)	95 (87.2%)	133 (89.3%)

Further analysis was performed to measure the association between the history of rifampicin treatment with the susceptibility rate of *C*. *albicans*. Table 5 shows that the proportions of *C*. *albicans* susceptible to fluconazole, ketoconazole, and itraconazole were lower (5.1%; 12.8%; and null respectively) among patients with history of rifampicin treatment compared to patients without history of rifampicin treatment (35%; 30%; and 5% respectively). There were significant associations between rifampicin exposure history and susceptibility of *C*. *albicans* to fluconazole (Odds Ratio: 23.00; 95% CI: 4.11–128.68; p <0.01) and ketoconazole (Odds Ratio: 7.17; 95% CI: 1.59–32.29; p <0.05), but not for itraconazole.

**Table 5:** Association of *C*. *albicans* susceptibility to fluconazole, ketoconazole, and itraconazole with patient’s rifampicin exposure.

Drug susceptibility	Rifampicin exposed patients (n = 49)	Rifampicin non-exposed patients (n = 10)	TOTAL
**Fluconazole**			
Susceptible	3(6.1%)	6 (60%)	9 (15.2%)
Resistant	46 (93.9%)	4 (40%)	50 (84.8%)
**Ketoconazole**			
Susceptible	6 (12.2%)	5 (50%)	11 (18.6%)
Resistant	43 (87.8%)	5 (50%)	48 (81.4%)
**Itraconazole**			
Susceptible	0 (0%)	1 (10%)	1 (1.7%)
Resistant	49 (100%)	9 (90%)	58 (98.3%)

**Fig 2:**
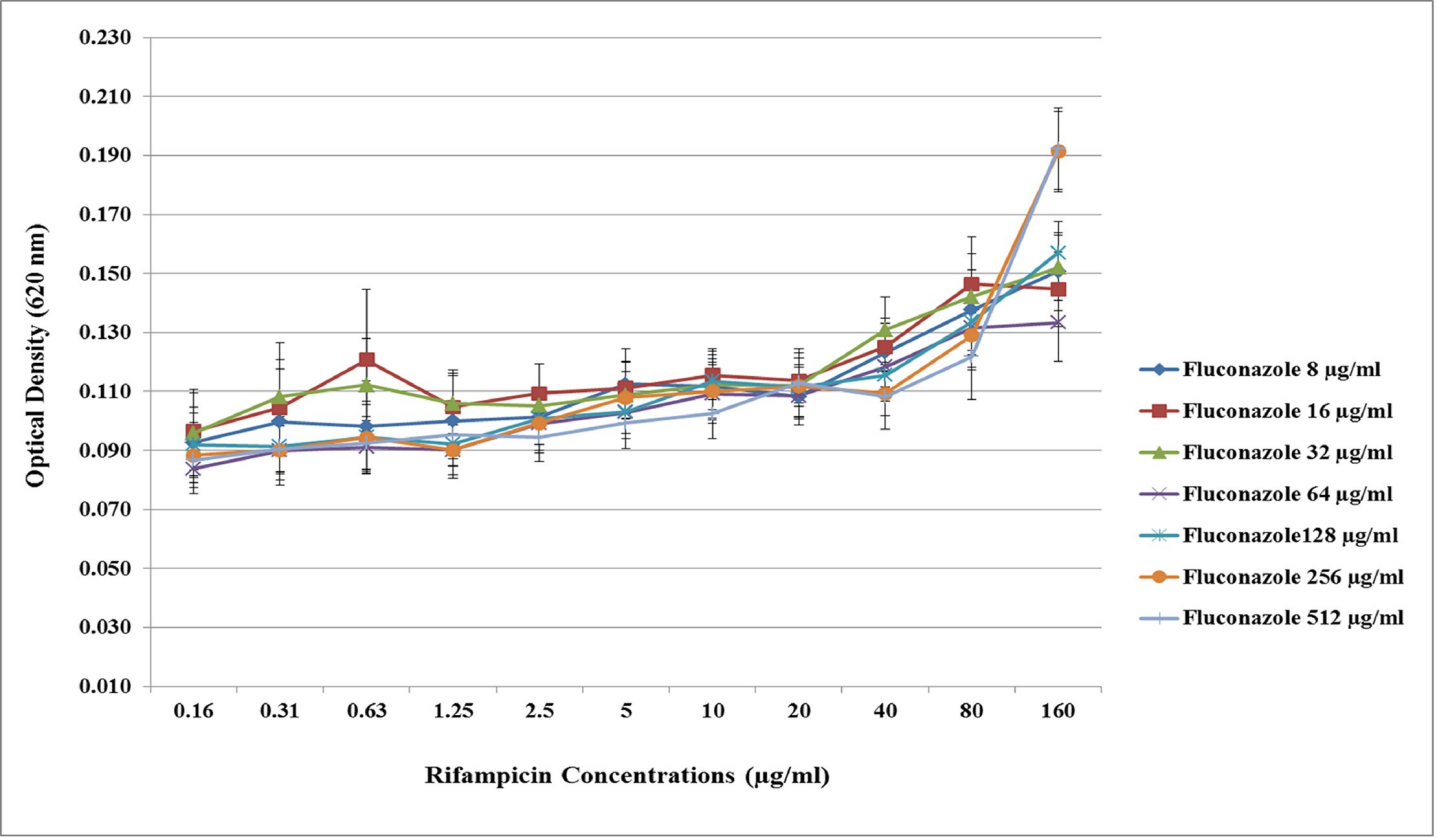
Effect of rifampicin to the fungicidal activity of fluconazole on the fluconazole resistant *C*. *albicans*. The Optical Density (OD) of the checkerboard microdilution assay results was measured. The colors of the line charts represent the different concentrations of fluconazole. The presence of rifampicin increased the OD of the cultured *C*. *albicans*, which correlates with the dose dependent increase in the population of viable yeast for all fluconazole concentrations. The ANOVA test showed significant difference in OD only for those with high fluconazole concentration (≥ 32 μg/ml) (p<0.05). The results were shown with the standard error of triplicate individual experiments of 8 fluconazole resistant *C*. *albicans* isolates.

**Fig 3:**
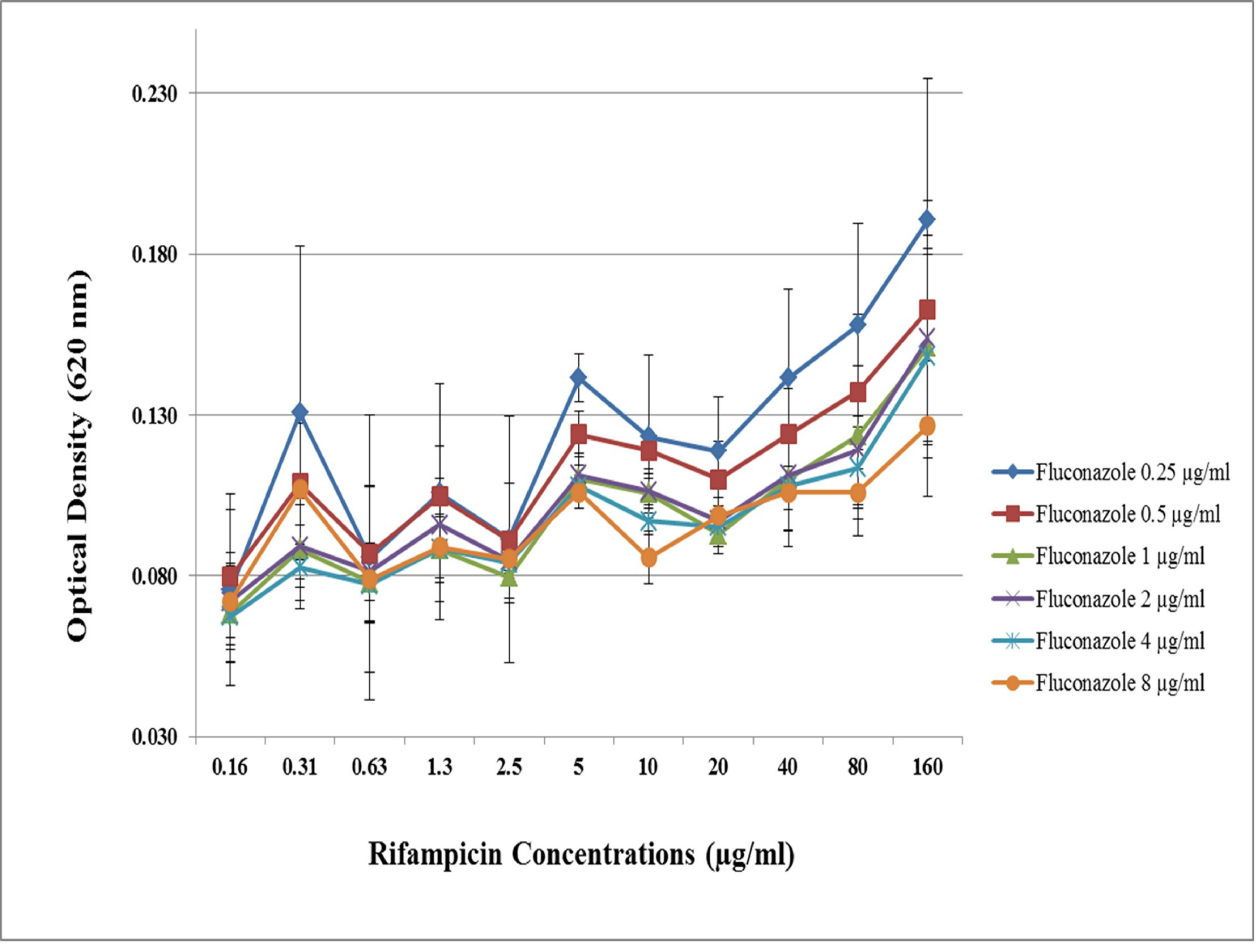
Effect of rifampicin to the fungicidal activity of fluconazole on the fluconazole sensitive *C*. *albicans*. The Optical Density (OD) of the checkerboard microdilution assay results was measured. The colors of line charts represent the different concentrations of fluconazole. The presence of rifampicin tended to increase the OD of the cultured *C*. *albicans* which correlates with the dose dependent increase in the population of viable yeast for all fluconazole concentrations. However, statistical analysis showed no significant difference (p>0.05). The results were shown with the standard error of triplicate individual experiments of 5 fluconazole sensitive *C*. *albicans* isolates.

Checkerboard microdilution assay was performed on 13 *C*. *albicans* isolates (8 fluconazole resistant and 5 sensitive) to test the effect of rifampicin on the fungicidal activity of fluconazole. The presence of rifampicin increases the OD of cultured *C*. *albicans* which correlates with the dose dependent increase in the population of viable yeast. The fungicidal effect of fluconazole was less effective because of rifampicin treatment. However, the effect of rifampicin was statistically significant only in the fluconazole resistant *C*. *albicans* isolates with high concentration of fluconazole treatments (≥ 32 μg/ml) (p<0.05) (Fig 2) but not in the fluconazole sensitive *C*. *albicans* with lower fluconazole concentration (Fig 3).

## Discussion

*Candida spp*. was found to be commonly colonized in the respiratory tract. The frequency of *Candida spp*. colonization was noted to be greater among non-tuberculosis individuals (Table 1). *Candida spp*. are opportunistic organisms whose clinical manifestation depends on the host’s immune response. As reported previously, *Candida spp*. colonization was influenced by the immune responses of the host [16, 17]. TB is a chronic infection that corresponds to the depletion of cellular immune response [25]. Although both TB and *Candida spp*. colonization are correlated with the depletion of cellular immune response, the infection process might involve different factors and mechanisms, resulting in the antagonism of both conditions.

A total of 71.6% patients were colonized by one species of *Candida spp*. However, if the patients were categorized as *M*. *tuberculosis* infected and non-infected, the proportion was significantly different. *M*. *tuberculosis* infected patients tended to have higher probability to be colonized by more than one species of Candida. *C*. *albicans* was still the most abundant species isolated from the sputum in both TB (42.5%) and non-tuberculosis (57.8%) patients. This finding is in line with previous reports. There are various studies that documented *C*. *albicans* as the predominant species isolated from patients with TB [6, 19, 26]. The results showed that the proportion of *C*. *albicans* was higher in non-tuberculosis patients compare to TB patients. The role of non albicans candida (NAC) in TB patients is not yet known. NAC was reported to have many important virulence factors that facilitate the colonization and invasion in the human body [27]. The combination with the deterioration of immune response in patients with TB might be the reason for the higher frequency of multi-species colonization and more frequent NAC colonization found to occur in patients with TB.

The proportion of *Candida spp*. susceptible to fluconazole was 38.3%, which is much lower compared to other reports [28–31]. The proportion of *Candida spp*. susceptible to ketoconazole was 10.7%, which is not different from a previous report (14%) by Khadka et al. in 2017 [31]. The proportion of *Candida spp*. susceptible to itraconazole was 1.3% which is significantly lower from previous reports (92.7%) [28, 32]. Remarkably, there was a high proportion of *Candida spp*. resistant to fluconazole, ketoconazole, and itraconazole that colonized the respiratory tracts of presumptive TB patients. This phenomenon was not reported in another series of patients with different clinical conditions.

The participants of this study sent their sputum samples to the laboratory because of suspicions of being infected with MDR-TB. Many of the results correlated with a history of ineffective management of the patients. It is therefore important to gather information of rifampicin administration prior to sputum examination in all cases. Rifampicin is one of the key drugs administered in the multidrug therapy approach of TB patients [1]. Rifampicin is a broad-spectrum antibiotic which was reported as having an antifungal effect on *C*. *albicans*. It has a synergistic effect with amphotericin B [33]. Rifampicin works by inhibiting the action of DNA-dependent RNA polymerases [34]. An important finding concerning the interaction of rifampicin with *C*. *albicans* is that exposure to rifampicin will induce the expression of *MDR1*, a gene that encodes a membrane transport protein of *C*. *albicans* which mediates resistance to fluconazole and other drugs. This upregulation of *MDR1* results in the increasing MIC of fluconazole [35]. Our data showed that rifampicin directly alters the fungicidal activity of fluconazole to *C*. *albicans* clinical isolates in the checkerboard microdilution assay. Rifampicin and fluconazole interactions were observed to be in dose-dependent manner which are consistent with the credible theory of *MDR1* gene upregulation. Taken together, the exposure of rifampicin among TB patients in this study might induce *C*. *albicans* resistance to fluconazole and ketoconazole. This possibility is the most plausible explanation for the high frequency of azole resistant *Candida spp*. among TB patients.

Rifampicin was reported to increase fibronectin binding to *C*. *albicans*. Recent experiments showed that fibronectin binding was strongly increased after rifampicin treatment indicating the induction of an additional factor contributing to increased adherence [36]. This mechanism might increase the possibility of *C*. *albicans* adherence in the respiratory tract of rifampicin exposed individuals, which may then increase the possibility of infection by *C*. *albicans*.

Our findings, which have never been reported, show the high prevalence of resistant *Candida spp*. colonization among presumptive MDR-TB patients. This report also highlights the possible influence of rifampicin, one of the most powerful anti-mycobacteria agents, on the susceptibility of *C*. *albicans* against fluconazole which is, the drug of choice for Candidiasis caused by *C*. *albicans*. This finding admonishes the prudent use of anti-mycobacteria in an endemic tuberculosis country like Indonesia. However, limitations of this study should be considered: (1) Rifampicin association with azole resistant of *C*. *albicans* (based on data taken from the criteria of sputum examination) reflects the history of rifampicin administration with a limited sample size. Although, the data was supported with the checkerboard assay result, the molecular mechanism and the actual interaction between the two drugs in the patients setting were not completely elucidated; (2) There is no further explanation of rifampicin interaction with NACs in the patients series.

## Conclusions

The study revealed that a high frequency of azole resistant *Candida spp*. colonized the respiratory tract of presumptive MDR-TB patients. The interaction of rifampicin with *C*. *albicans* may indicate an association of chronic exposure to rifampicin with induction of azole resistance.
